# The Emergence of Explicit Knowledge in a Serial Reaction Time Task: The Role of Experienced Fluency and Strength of Representation

**DOI:** 10.3389/fpsyg.2017.00502

**Published:** 2017-04-04

**Authors:** Sarah Esser, Hilde Haider

**Affiliations:** General Psychology 1, Department of Psychology, University of CologneCologne, Germany

**Keywords:** implicit learning, conscious awareness, fluency, associative strength, serial reaction time task, unexpected events

## Abstract

The Serial Reaction Time Task (SRTT) is an important paradigm to study the properties of unconscious learning processes. One specifically interesting and still controversially discussed topic are the conditions under which unconsciously acquired knowledge becomes conscious knowledge. The different assumptions about the underlying mechanisms can contrastively be separated into two accounts: single system views in which the strengthening of associative weights throughout training gradually turns implicit knowledge into explicit knowledge, and dual system views in which implicit knowledge itself does not become conscious. Rather, it requires a second process which detects changes in performance and is able to acquire conscious knowledge. In a series of three experiments, we manipulated the arrangement of sequential and deviant trials. In an SRTT training, participants either received mini-blocks of sequential trials followed by mini-blocks of deviant trials (22 trials each) or they received sequential and deviant trials mixed randomly. Importantly the number of correct and deviant transitions was the same for both conditions. Experiment 1 showed that both conditions acquired a comparable amount of implicit knowledge, expressed in different test tasks. Experiment 2 further demonstrated that both conditions differed in their subjectively experienced fluency of the task, with more fluency experienced when trained with mini-blocks. Lastly, Experiment 3 revealed that the participants trained with longer mini-blocks of sequential and deviant material developed more explicit knowledge. Results are discussed regarding their compatibility with different assumptions about the emergence of explicit knowledge in an implicit learning situation, especially with respect to the role of metacognitive judgements and more specifically the Unexpected-Event Hypothesis.

## Introduction

Implicit learning refers to our ability to adapt to more or less complex statistical structures in the environment without possessing conscious access to the content of the acquired knowledge, often even lacking any conscious knowledge that something has been learned at all. An accessible example is the knowledge about grammatical rules which develops early and is hard to verbalize even for adults (Reber, 1989; Dienes et al., 1991). But implicit learning processes are also important for sequences of movements (e.g., typing on a keyboard; Nissen and Bullemer, 1987; Willingham, 1998) or stimuli (e.g., visual, spatial, or auditive; Haider et al., 2012; Ling et al., 2016), as well as for social interactions (Heerey and Velani, 2010; Norman and Price, 2012).

There are two very prominent paradigms for studying these so-called implicit learning processes: One is the Artificial Grammar Learning Task (AGLT; Reber, 1989) and the other one is the Serial Reaction Time Task (SRTT; Nissen and Bullemer, 1987). In an AGL task participants are confronted with letter strings that are constructed from artificial probabilistic grammar rules. In a following test task participants are usually asked to classify test strings as following or not following this rule. In a SRTT participants react to a series of different target stimuli by choosing a corresponding response. Unbeknownst to the participant the stimuli and/or the responses follow a certain probabilistic or deterministic sequence. Test tasks can, mostly depending on the definition of consciousness, range from recognition (Shanks and Johnstone, 1999), over free generation (Wilkinson and Shanks, 2004), process dissociation (Jacoby, 1991; Destrebecqz and Cleeremans, 2001), and subjective measures (Persaud et al., 2007; Haider et al., 2011) to verbal report (Eriksen, 1960; Rünger and Frensch, 2010). In both paradigms, the SRTT and the AGLT, participants are usually unaware of the fact that their task followed a certain rule or sequence, while their performance shows that, in fact, they have acquired some knowledge about these [see e.g., Rünger and Frensch, 2010, for a discussion about the adequacy of the different measures for (un-)conscious knowledge].

In implicit learning research, a lot of effort is dedicated to the question about the complexity, the flexibility and the structure of the knowledge we extract from predictable structures (see e.g., Abrahamse et al., 2010, for an overview). Here, our goal is to focus not on implicit learning processes *per se*, but on the question: Why and how do some participants acquire conscious knowledge about the unconsciously learned sequences? The paradigms used in implicit learning research can constitute very useful tools for addressing exactly this important topic.

One undoubtedly important aspect that divides unconscious from conscious information processing is signal strength (Kanwisher, 2001; Cleeremans and Jiménez, 2002). While research on subliminal priming mostly leads to the conclusion that perception seems to be unconscious when the signal strength is very weak, there is more disagreement about whether having a high signal strength is a sufficient condition for a bottom-up signal to become conscious (Dehaene et al., 2006). Depending strongly on the definition of consciousness and a potential division between phenomenal and access consciousness (Block, 2005; Kouider et al., 2010; Cohen and Dennett, 2011), some claim that strong activity in specialized local circuits (e.g., in extrastriate areas; Lamme, 2003, 2010; Zeki, 2003; Block, 2007) is sufficient and, thereby, allowing different, gradual qualities of conscious perception (Overgaard et al., 2006; Nieuwenhuis and de Kleijn, 2011; Windey et al., 2014). Other researchers assume that a global, cortical “ignition,” involving parieto-frontal networks which allow for a top-down amplification (Dehaene and Naccache, 2001; Dehaene et al., 2003; Lau and Passingham, 2006; Dehaene and Changeux, 2011), is correlated with conscious information processing. In this latter class of theories, consciousness is usually seen as an all-or-none matter (Sergent and Dehaene, 2004; Kouider et al., 2010). While opinions still strongly differ about the definition of conscious processing, there is more agreement that global processing is associated with reportability and highly flexible, strategic usage of the respective knowledge (Block, 2005; Lamme, 2010; Cohen and Dennett, 2011). When we further speak of conscious or explicit knowledge in this article, we refer to this highly flexible, reportable form of knowledge.

The debate about the role of bottom-up signal strength and additional higher-order top-down processes is also reflected in the proposed mechanisms for the transformation from implicit to explicit knowledge which shall further be the subject of this paper. While there certainly are more diverging theories than can be discussed here in detail, most of them can roughly be differentiated by the role of the strength of associative weights of the implicitly learned structure. Some theories, implying a quantitative, gradual difference, assume that representational strength and stability is the deciding factor for separating unconscious from conscious processing. By contrast, others assume qualitative differences where additional, higher order processes are necessary to transfer unconscious into conscious knowledge.

The former of these accounts is the more parsimonious one. No qualitative separation between conscious and unconscious representation is assumed, but rather a gradual transition. These theories do not need any additional, hierarchically higher, mechanisms that transform unconscious into conscious representations. Therefore, these models are often referred to as *single-system views*. One and the same learning, respectively memory system can explain fast reaction times or simple discriminative decisions when its associations are relatively weak and, as these grow stronger throughout training, can account for verbally accessible and highly flexible knowledge (Cleeremans and Jiménez, 2002; Perruchet and Vinter, 2002; Shanks and Perruchet, 2002; Destrebecqz and Cleeremans, 2003).

In contrast to these theories, there are so-called *multiple-system views* which assume a qualitative distinction between unconscious and conscious representations. According to these accounts, representational strength is not enough to explain the differences between both forms of knowledge. Instead, consciousness is defined by a distinct form of information processing. Therefore, it either has to be explained how unconscious information is granted access to this particular form of processing, potentially involving top-down amplification and global processing (Keele et al., 2003), or how an independent learning system builds explicit knowledge on its own (Reber, 1989; Willingham, 1998; Sun et al., 2001; Frensch et al., 2003; Haider and Frensch, 2005; Scott and Dienes, 2010).

There are hierarchical models that build a bridge between stricter single-system and multiple-systems views. An interesting account that fits into this position comes from Cleeremans et al. (Cleeremans, 2011; Timmermans et al., 2012). Similar to single-system views, they regard the gradually developing strength, stability, and distinctiveness of representations as necessary conditions for gradually developing explicit knowledge. Nevertheless, they also include *Higher-Order Thought (HOT) Theories* of consciousness (Rosenthal, 1997; Lau, 2008) into their theory. HOT Theories assume that consciousness develops if a hierarchically built system is able to represent that it knows or does not know something (metacognition). In Cleeremans (2011) theory, a simple feed-forward backpropagation network develops first-order knowledge about any sequential structure which enables predictive behavior. A similarly built, hierarchical higher network simultaneously learns about the states of the first-order system when this has made a correct or an incorrect prediction and thereby develops second-order knowledge about the first-order system possessing or not possessing knowledge in a given situation. The assumption that the higher order mechanism operates in the same way as the first order mechanism makes this model a hybrid between single- and multiple-system views. While multiple hierarchical layers of learning are required for consciousness to develop, the same strengthening mechanism is assumed for the generation of implicit and explicit knowledge. Consciousness about the implicitly learned sequence is still a gradual state, changing with every trial from not-knowing to knowing whether something is known, depending on the strength of the associative weights in the higher-order network.

Metacognition also plays an important role in other multiple-system views about the transition from implicit to explicit knowledge. In the *Unexpected Event Hypothesis* (UEH), implicitly learned contents are assumed to be encapsulated in local modules. They can neither become conscious themselves just by increasing strength throughout the learning process, nor does the explicit learning process have direct access to the implicit information. Instead, an indirect link is assumed, by which explicit knowledge can develop when implicit learning leads to observable, consciously perceivable, unexpected changes in a person's behavior (Frensch et al., 2003; Haider and Frensch, 2005, 2009; Rünger and Frensch, 2008). For example, having learned a motor sequence implicitly in an SRT task might lead to premature responses before the next target stimulus appears (Haider and Frensch, 2009). This in turn might surprise the participant, who might then start an attributive search process about the reason for their ability to respond prematurely which often leads to the detection of the underlying sequence. Still, as long as there are other more obvious options the unexpected change in behavior might be attributed to, search processes might be terminated and no explicit knowledge might develop (Haider and Frensch, 2005). Importantly, while the implicit learning process is assumed to develop knowledge by a rather slow strengthening of associative weights (Cleeremans and Dienes, 2008), the explicit learning mechanism does not operate in the same way. Instead of accumulating strength via feedback about the correctness of predictions, explicit attributional search processes result in sudden insights following the unexpected occurrence of predictive behavior. In an SRT task these sudden insights are characterized by an abrupt drop in the reaction times (Haider and Rose, 2007; Haider et al., 2011) as well as an increased coupling of gamma-band activity between the right prefrontal and occipital regions (Rose et al., 2010; Wessel et al., 2012). Hence, according to the UEH, consciousness about the sequence is not seen as a gradual matter, relying on a slow strengthening of associations on a trial-by trial basis, as proposed by single-system views or Cleeremans' hybrid model (Cleeremans, 2011). Nevertheless, the UEH would not dispute the idea that there are higher-order learning processes which develop knowledge about first-order states and are relevant for assessing what the system knows or does not know. These learning processes might for example be important for the accuracy of judgements of knowledge. Increasing accuracy of judgements of knowledge might in turn serve as a consciously perceivable, unexpected change in behavior, which again leads to conscious sequence knowledge. In the UEH any metacognitive judgement about one's own behavior (perceiving increased fluency, accuracy, speed etc. or unexpected mismatches in these aspects when the sequential structure is suddenly replaced with irregular trials) can serve as an unexpected event, triggering attributive processes.

Scott and Dienes (2008, 2010) proposed a similar account with implicit learning leading to *explicit judgement knowledge* (gradual improvement of the accuracy of second-order judgements). Experiencing this meta-knowledge about one's own behavior can trigger a second attributional process leading to *explicit structure knowledge* (insight into the sequential rules). Within the AGL paradigm, they have shown that participants first notice an increasing correlation between their feeling of familiarity for learned grammar strings and their ability to discriminate learned from new grammar strings (explicit judgement knowledge). Perceiving this surprising ability, a second explicit learning process is triggered, leading to explicit structure knowledge of the grammatical rules.

It is beyond the scope of this paper to discriminate between the finer differences of these proposals. Rather, the aim is to offer some insights into the fundamental assumptions about the role of the strengthening of associative weights for the transition from implicit to explicit knowledge. So far, not many studies have empirically explored whether the same associative strength can result in different development of explicit sequence knowledge. While single-system, respectively strengthening accounts would not expect such a difference in explicit knowledge, multiple-system accounts like the UEH would allow different extents of explicit knowledge for the same associative strengths, as long as the learning situations lead to different metacognitive judgements of one's own behavior.

### Rationale of the experiments

In order to test the role of the strengthening of associative weights against the role of metacognitive knowledge, we aimed to create a situation where the associative strength is kept constant between two conditions but the metacognitive judgements, or more specifically here, the subjective feelings of fluency differ. We assume that participants who have the opportunity to experience unexpected differences in their feeling of fluency are likely to use this feeling as a trigger for explicit search processes, ultimately resulting in more explicit sequence knowledge.

For testing this, we used an SRTT with the important modification of the arrangement of trials that follow the sequence (regular trials) and those that violate the sequence (deviant trials). More precisely, we aimed to keep the number of correct and incorrect transitions throughout the experiments equal between all conditions. However, in one condition the regular trials and deviant trials were arranged in alternating mini-blocks, while they were arranged randomly in the other condition. It is assumed that, due to the equal number of correct and incorrect transitions, all conditions should be able to develop the same strength of associative weight and gain a comparable rate of implicit knowledge (Experiment 1). Concurrently, the difference in the arrangement of trials should lead to differences in the experienced fluency of the task. Participants working with mini-blocks of regular and random trials should tend to experience fluency differences between regular and random material, while participants with randomly arranged material should not experience such differences (Experiment 2). Participants who experience differences in the fluency of the task should have a tendency to search for the reasons of these experienced differences and hence show a greater likelihood to develop explicit sequence knowledge (Experiment 3). All experiments used the same method.

## General method

### Stimulus and apparatus

**Figure 1:**
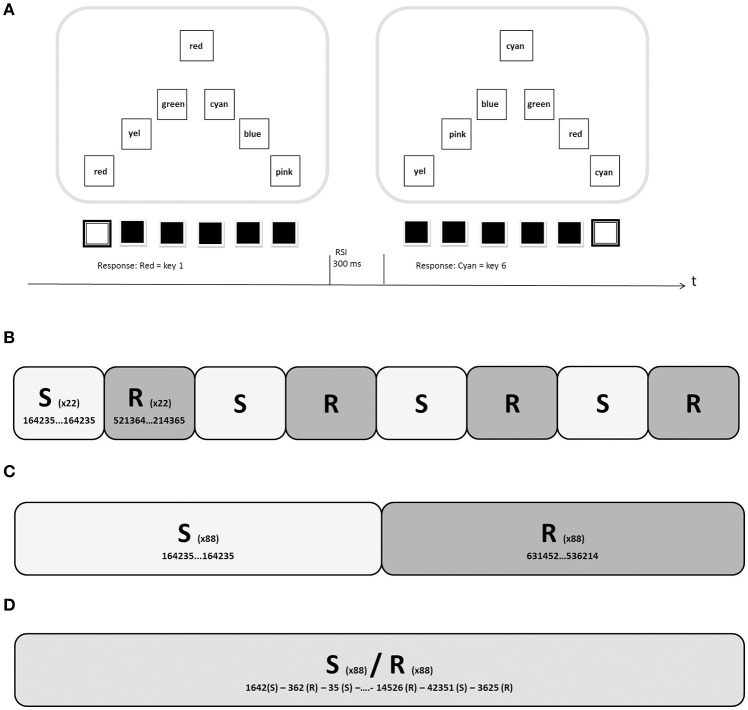
**(A)** Two trials in the SRTT training (simple colored Stimuli; Experiment 2). **(B)** Structure of one block of the BO-Condition in Experiments 1 and 2. Twenty-two sequential trials (S) were followed by 22 random trials (R), resulting in 176 total trials per block. Experiment 1 consisted of seven blocks, Experiment 2 of six blocks. **(C)** In the BO-Condition of Experiment 3, 88 sequential trials were followed by 88 random trials (or vice versa) in each block. It was balanced in all experiments whether a block started with sequential or random material. **(D)** One block of the RO-Condition in all three experiments consisted of 88 random and 88 sequential trials mixed randomly.

In our version of the SRTT (Haider et al., 2012), a squared target stimulus appeared in the upper third of the screen. The target stimulus had a size of about 3 × 3 cm on a 17-inch screen and contained a picture of one of six different colored circles. In the lower two thirds of the screen six squared response stimuli were presented in a triangular space, with each stimulus having a size of about 2.8 × 2.8 cm. On each trial, the response stimuli displayed the six possible target stimuli. The arrangement of the six possible target stimuli within the response squares was changed from trial to trial (Figure 1A). The locations of the response stimuli were spatially mapped to the Y, X, C, B, N, and M keys of a German QWERTZ-keyboard. The participants were instructed to let their index-, ring-, and middle-fingers rest on the six keys for the entire experiment and to press the key that spatially corresponded to the response stimulus which contained the target stimulus.

In all three experiments, 50% of the locations of the correct response stimulus followed a regular sequence, which resulted in a motor sequence of key strokes for the participants. Described as response positions 1–6, the resulting sequence was 1-6-4-2-3-5. The colored target stimuli were presented randomly with the constraints that all six possible targets had occurred before being shown again and that no response stimulus position would contain the same stimulus more than twice consecutively. In the other 50% of the trials, both the colored target stimuli and the response stimulus locations were presented randomly, with the constraints that no position would contain the target stimulus successively and that all six positions were to be used equally often in each of the training blocks. The crucial manipulation in all three experiments was the arrangement of the regular and random trials. In the *Blocked-Order Condition* (BO-Condition), 22 regular trials were always followed by 22 random trials in the first two experiments (Figure 1B). In Experiment 3, the length of these mini-blocks was extended to 88 trials (Figure 1C). In the *Random-Order-Condition* (RO-Condition) random and sequential trials were mixed randomly (Figure 1D). All participants received the same material, but the starting point was assigned randomly for each participant.

There were very slight differences in the proportion of regular and deviant trials in both conditions. While the BO-Condition had 49% regular and 51% deviant trials, the RO-Condition contained 53% regular and 47% deviant trials in all experiments. If these small differences, against our intention and expectation, made a relevant difference for the associative strengths, then the RO-Condition would build stronger associations, which would not be in favor of our hypotheses.

### Procedure

Each experiment consisted of a training and a test phase. The training started with the presentation of the instructions on the computer screen. Participants were instructed to react as fast as possible while also avoiding mistakes. They were informed that they would first receive 20 practice trials to get a first impression of the task. These practice trials consisted of random material that followed the same constraints as the random material described above. Each trial of the task began with the presentation of the six response stimuli. After 100 ms the target stimulus appeared for 150 ms. After the participant's response the screen went black for 300 ms before showing the next six response stimuli in a different arrangement. The course of a trial was identical in the test phases. After the respective test, participants were debriefed and received their financial reward or their course credit.

## Experiment 1

The aim of Experiment 1 was to show that the extent of implicit knowledge depends on the amount of regular transitions presented during training, not on the respective arrangement of regular and random trials. As it is conceivable that the different arrangements of the training material cause participants to behave differently in the training tasks, even though they possess comparable amounts of knowledge, we assessed the extent of acquired knowledge with two different test tasks after training. Half of the participants were tested with a wagering task (Persaud et al., 2007; Haider et al., 2011). The other half received additional test blocks in which they were confronted with blocks of either sequential or random trials only. With the use of two different test tasks we aimed to assess different aspects of implicit learning. The wagering task assesses implicit knowledge without resorting to reaction times by asking participants to predict the next response. By additionally asking participants to wager on the correctness of their guess, this test enables us to detect whether participants possess explicit knowledge. The three additional test blocks in the second test are especially important as they assess knowledge in terms of the usual performance differences between random and regular blocks. Thus, it should detect knowledge with the same sensitivity as the training phase (Shanks and St. John, 1994). This would allow us to test whether any possible training differences between the two conditions reflect different amounts of knowledge or different expressions of a comparable extent of learning. If it is only the amount of regular transitions that influences learning, the two conditions should not differ in the two tests. By contrast, if the arrangement of trials matters, then the two conditions should differ in the two test tasks.

### Method

#### Participants

One hundred-twenty students of the University of Cologne (81 women) participated either in fulfillment of course credit or in return for payment, with 60 participants in the Blocked-Order and 60 participants in the Random-Order Condition which they were randomly assigned to. The mean age was 24.4 years (range: 18–47, *SD* = 5.28). All participants reported normal or corrected-to-normal vision.

#### Procedure

The procedure of the SRTT training was as described in the General Method Section. The training task consisted of seven blocks with 176 trials (4 × 22 sequential trials alternating with 4 × 22 random trials in the BO-Condition).

For 60 participants (30 in each condition) the training ended after these seven training blocks and they received the instructions for the following wagering task. The wagering task had the same design as the training task with two important exceptions. First, it only contained regular trials. Second, on 36 of the 176 trials, the target stimulus was replaced with a question mark. Participants were instructed to respond with the key they considered to be the most likely response after the previous trial. The question mark remained on the screen until the participant gave a response. Subsequently, they were asked to indicate how sure they were about the correctness of their prediction by wagering either 1 or 50 Cent on their response. They were informed that they would earn the amount if their prediction was correct and to lose it if it was incorrect. Following the logic of the zero-correlation criterion (Dienes and Perner, 1999; Dienes, 2008), this task allows to assess whether participants possess implicit or explicit knowledge about the sequence. Participants with implicit knowledge give more correct answers than to be expected by mere guessing, while showing no correlation between the correctness of their response and their confidence (the amount wagered). By contrast, participants with explicit knowledge should demonstrate a high rate of correct responses that is above chance-level and a strong correlation between the correctness of their response and their confidence.

For 60 participants (30 in each condition) the seven training blocks were followed, without any special notice or new instruction, by three test blocks. Their only difference to the training blocks was that the first and last test block contained purely random trials, whereas the second block consisted of solely regular trials.

### Results

We had to exclude six participants due to a technical error with one computer (four in the BO-Condition). For the remaining 114 participants we analyzed the mean error-rate for each participant. Participants were excluded if their error rate, averaged over all seven blocks, exceeded 15%. This led to the exclusion of four participants in the BO-Condition and six participants in the RO-Condition. Also one person of the BO-Condition had to be excluded, because they did not finish the test task. For the remaining 51 participants in the BO-Condition and 52 participants in the RO-Condition, individual median reactions times (median RTs) were computed for each block. We excluded errors and post-error trials from this computation (10.027%). We excluded post-error trials because of post-error slowing (Ruitenberg et al., 2014) and because trials after an erroneous response also are based on a wrong transition. Also RTs larger than 3000 ms (0.346%) were excluded.

#### Training phase

Tables 1A,B depict the mean percent error rates and the means of the median RTs of the training. For both dependent variables, we conducted a 2 (Condition) × 2 (Trial Type: regular vs. deviant trial) × 7 (Block) mixed-design ANOVA.

**Table 1A:** **Percent error rates and their respective standard deviations (in brackets) by training block, condition, and trial type (regular vs. deviant) in Experiment 1**.

**Training Block**	**Random-order condition**	**Random-order condition**	**Blocked-order condition**	**Blocked-order condition**
	**Regular trials**	**Deviant trials**	**Regular trials**	**Deviant trials**
Block 1	8.50 (4.32)	9.57 (5.89)	6.44 (5.55)	7.33 (4.12)
Block 2	6.62 (4.68)	8.21 (4.37)	4.40 (3.29)	6.07 (3.35)
Block 3	4.85 (3.41)	6.45 (5.04)	4.11 (3.21)	5.55 (3.64)
Block 4	4.47 (2.88)	5.93 (3.86)	3.94 (3.31)	4.84 (3.42)
Block 5	5.05 (4.03)	6.48 (4.40)	3.06 (2.05)	5.41 (3.50)
Block 6	4.98 (3.84)	5.55 (3.78)	3.07 (3.14)	5.75 (4.80)
Block 7	4.70 (5.50)	6.80 (4.86)	4.05 (3.02)	5.55 (4.10)

**Table 1B:** **Means of the median RTs and their respective standard deviations (in brackets) by training block, condition, and trial type (regular vs. deviant) in Experiment 1**.

**Training Block**	**Random-order condition**	**Random-order condition**	**Blocked-order condition**	**Blocked-order condition**
	**Regular trials**	**Deviant trials**	**Regular trials**	**Deviant trials**
Block 1	995.2 (199.8)	1002.0 (190.4)	991.4 (185.3)	998.4 (185.0)
Block 2	928.0 (150.3)	954.5 (156.4)	917.5 (126.2)	938.8 (115.7)
Block 3	982.3 (132.7)	907.7 (128.6)	887.9 (103.2)	891.9 (106.8)
Block 4	865.5 (121.2)	886.7 (121.8)	846.4 (96.3)	875.9 (101.8)
Block 5	848.2 (111.5)	864.9 (108.2)	815.0 (83.5)	857.9 (94.7)
Block 6	829.3 (110.8)	857.8 (118.9)	807.5 (88.5)	850.4 (85.6)
Block 7	805.2 (96.7)	834.0 (98.62)	786.9 (83.7)	830.2 (91.4)

For mean error rates, a 2 (Condition) × 2 (Trial Type: regular vs. deviant trial) × 7 (Block) repeated measures ANOVA yielded a main effect of Block [*F*_(6, 606)_ = 23.77, *p* < 0.001, η_*p*_^2^ = 0.190] and of Trial Type [*F*_(1, 101)_ = 76.18, *p* < 0.001, η_*p*_^2^ = 0.430]. As can be seen from Table 1A, error rates decreased over the course of training and error rate was higher on deviant trials. There also was a main effect of Condition [*F*_(1, 101)_ = 5.57, *p* = 0.020, η_*p*_^2^ = 0.052], due to the RO-Condition making more errors (6.3% for the RO-, 4.9% for the BO-Condition). The interaction did not reach the level of significance.

For median RTs a 2 (Condition) × 7 (Block) × 2 (Trial Type: regular/deviant) mixed-design ANOVA revealed a significant main effect of Block [*F*_(6, 606)_ = 140.33, *p* < 0.0001, η_*p*_^2^ = 0.581] and of Trial Type [*F*_(1, 101)_ = 138.34, *p* < 0.0001, η_*p*_^2^ = 0.578]. Table 1B shows that participants in both conditions became faster over the course of the training and responded faster to regular than to random trials. There also was a significant Block × Trial Type interaction [*F*_(6, 606)_ = 4.34, *p* < 0.001, η_*p*_^2^ = 0.041], indicating that the differences between regular and deviant trials increased over the course of the training, representing a general learning effect. Follow-up interaction contrasts (Block 1 vs. 7 × Trial Type) revealed that both conditions showed a sequence learning effect [*F*_(1, 101)_ = 5.41, *p* = 0.022, η_*p*_^2^ = 0.051 for the RO-Condition and *F*_(1, 101)_ = 9.60, *p* = 0.003, η_*p*_^2^ = 0.087 for the BO-Condition]. No other interaction reached the level of significance. There was a trend toward a significant Condition × Trial Type interaction [*F*_(1, 101)_ = 3.07, *p* = 0.083, η_*p*_^2^ = 0.030]. This trend might be due to a somewhat larger overall difference between regular and deviant trials in the BO-Condition than in the RO-Condition. Further, *post-hoc* tests point to the circumstance that the larger difference between regular and deviant trials within the BO-Condition is only present in the last three [*F*_(1, 101)_ = 8.49, *p* = 0.004, η_*p*_^2^ = 0.078] but not in the first three blocks (*F* < 1). Taken together the trend toward a Condition × Trial Type interaction might indicate that, against our assumptions, there could be a difference in the extent of acquired knowledge between the two conditions with the BO-Condition acquiring more knowledge than the RO-Condition.

#### Test phase: wagering task

We first tested whether the participants' acquired sequence knowledge is better than expected by chance. For this purpose, we tested the proportion of correct predictions in each condition against a chance level of 20% (which implies that participants only know that the same button is never to be pressed successively). In both conditions the proportion of correct predictions was significantly higher than chance level [*t*_(26)_ = 2.70, *p* = 0.006, one-tailed, for the RO-Condition; *t*_(24)_ = 2.54, *p* = 0.009, one-tailed, for the BO-Condition; see Table 2]. This further confirms that both conditions acquired knowledge about the sequence which has already been shown in the reaction times of the training phase. Furthermore, the amount of knowledge did not differ between conditions [*t*_(50)_ = 0.70, *p* = 0.487].

**Table 2:** **Percent correct predictions, percent high wagers given when the prediction was correct, percent high wagers given when the prediction was false, and their respective standard deviations (in brackets) by condition in Experiment 1**.

**Condition**	**Percent correct predictions**	**Percent high wager| correct prediction**	**Percent high wager| false prediction**
Random-order	25.65 (11.00)	55.72 (34.94)	58.84 (32.35)
Blocked-order	28.50 (16.43)	48.50 (28.16)	46.31 (30.56)

Since our aim of the experiment was to show that the RO- and the BO-conditions did not differ with regard to the strength of their representations of the regular sequence, our main focus was on this Null-hypothesis testing. Therefore, we additionally conducted a Bayes analysis. Following Dienes (2014) we specified our roughly expected maximum effect-size if the hypothesis was true that both conditions differ. Based on the data of Experiment 3 in this paper and data from previous studies (Haider et al., 2011, 2012, 2014) we estimated that the maximum expected difference between the percent correct predictions in the BO- and the RO-Condition can reach 30% [presented as *B*_*H*__(0.30%)_]. With this estimated effect size the Bayes factor was *B*_*H*__(0.30%)_ = 0.02. According to the conventions of Jeffreys (1939), a *B* smaller than 1/3 can be taken as substantial evidence for the H_0_ (a *B* > 3 is taken as substantial evidence for the H_1_). Taken together, these results confirm that both conditions acquired knowledge about the sequence and that the amount of acquired knowledge did not differ between both conditions.

Next, we investigated whether this knowledge was explicit. If so, participants should be able to strategically use their knowledge to maximize their gains. We compared the proportion of high wagers when participants made correct predictions with the proportion of high wagers when they made a false prediction (see Table 2). A 2 (Condition) × 2 (Wager Type: high|false vs. high|correct) mixed-design ANOVA yielded no significant effect (all *F*s < 1). Hence, it seems that both groups acquired a comparable amount of merely implicit sequence knowledge.

To exclude that the participants acquired explicit knowledge during the test task, we contrasted the difference between high|correct and high|false wagers in the first 12 wager trials with that of the last 12 wager trials for both conditions. No Condition showed a significant increase in the difference between high|correct and high|false wagers (both *t*s < *1*).

#### Test phase: transfer blocks

For the error rates, a 2 (Condition) × 2 (Test Block 8/10 vs. 9) mixed-design ANOVA revealed a significant effect of Block [*F*_(1, 49)_ = 18.93, *p* < 0.001, η_*p*_^2^ = 0.279] indicating less errors in the regular than in the random blocks. Neither the main effect of condition (*F* < 1) nor the Condition × Block interaction reached the level of significance [*F*_(1, 49)_ = 2.79, *p* = 0.101, η_*p*_^2^ = 0.054].

**Figure 2:**
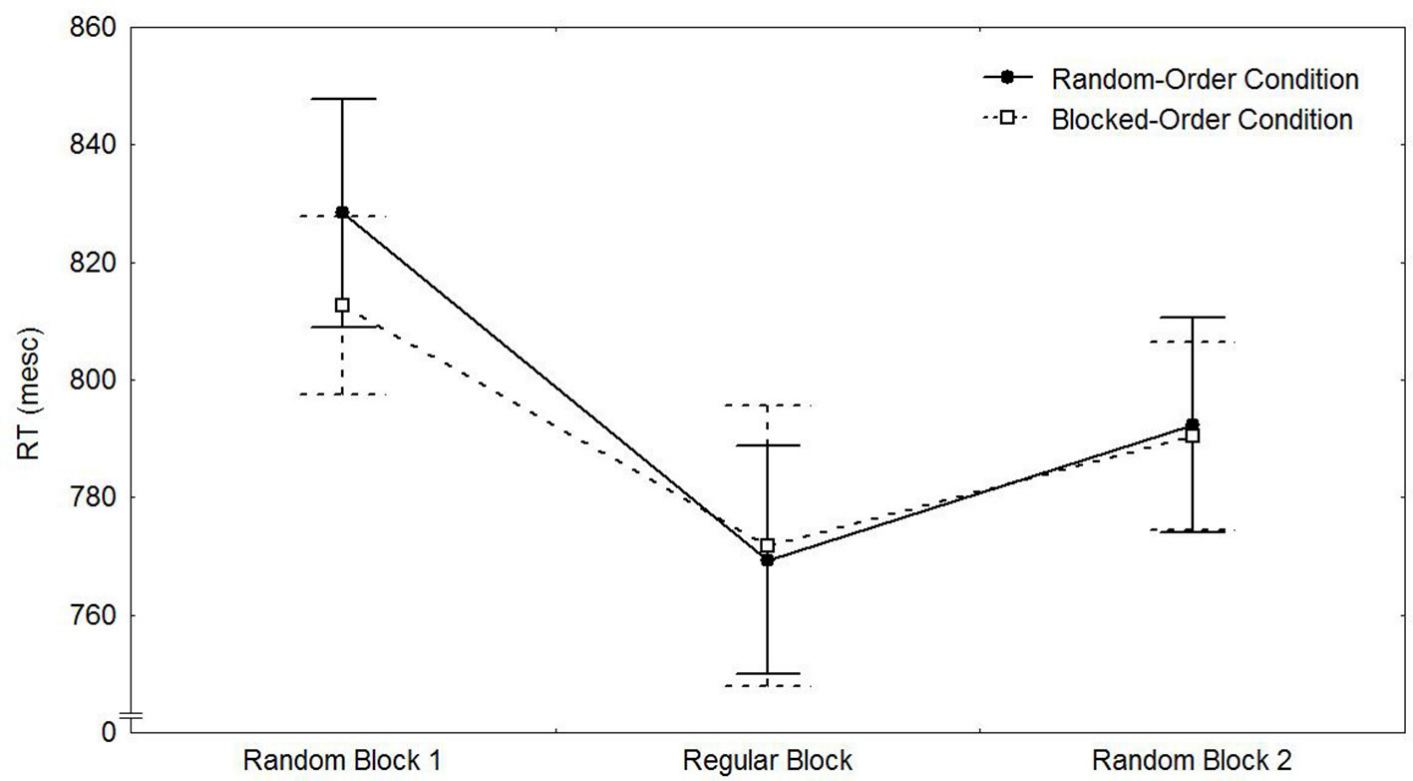
**Means of the median RTs and their respective standard errors by transfer block and condition in Experiment 1**.

Figure 2 shows the mean median reaction times of both conditions for the test phase. A 2 (Condition) × 2 (Block Type 8/10 vs. 9) mixed-design ANOVA only revealed a significant main effect for Block [*F*_(1, 49)_ = 17.84, *p* < 0.001, η_*p*_^2^ = 0.267]. As can be seen from Figure 2, participants in both conditions responded slower in the random blocks than in the sequential block.

In order to test whether the data of the transfer test speak for our null-hypothesis that both conditions do not differ in their acquired knowledge we again used Bayes statistics. Therefore, we needed to specify the maximum plausible effect for the difference between regular and deviant transfer test blocks for both conditions. If both conditions did differ in their acquired knowledge, the BO-Condition should show a greater difference than the RO-Condition. We set 50 ms as the maximum plausible difference between the difference of regular and deviant trials in the RO-, respectively, the BO-Condition. These 40 ms were taken as an estimation because of (a) the data from the last three training blocks of Experiment 3 and (b) the data of two Experiments of Eberhardt et al. (manuscript submitted for publication). The former was chosen because there we predicted a difference in the amount acquired knowledge. In the latter two, participants were also trained with material arranged similar to our RO-Condition and later tested with a comparable transfer test, with the difference that we predicted and found a difference in the amount of acquired knowledge due to different instructions. With this estimated effect size the Bayes factor was *B*_(0.40 *ms*)_ = 0.14. A *B* < 1/3 indicates that the data of the transfer block are in favor of the null-hypothesis. Thus, both conditions do not seem to differ in the amount of the participants' acquired knowledge.

### Discussion

The results of Experiment 1 show that participants in both conditions acquired sequence knowledge during training, indicated by the decrease of RTs and the significant Block × Trial Type interaction. However, there was a statistical trend in the Condition × Trial Type interaction with the BO-Condition showing a somewhat larger difference between regular and deviant trials than the RO-Condition, particularly in the last three training blocks. At first glance, this might contradict our hypothesis that the amount of correct and incorrect transitions determines the extent of acquired sequence knowledge, not the arrangement of these trials.

However, when, in the two test phases, participants in both conditions were given the same chance to express their knowledge the results cast a different impression on the data. The wager task shows that both conditions acquired knowledge about the sequential structure. More importantly, it also reveals no difference in the amount of acquired knowledge between the two conditions as they made a comparable amount of correct predictions. The data of the transfer test also support this finding. Both conditions show similar differences between regular and deviant trials and neither the effect of Condition nor the Condition × Block interaction was significant. This is especially important as it might be argued that the wager task is less sensitive and therefore not able to detect small differences in participants' knowledge (Shanks and St. John, 1994). The transfer test gives the participant the possibility to express their knowledge in the exact same way as they did in the training phase. This further fortifies the impression that both conditions acquired a comparable amount of knowledge.

Thus, it seems likely that the differences between conditions found in the training task do not indicate a difference in the amount of acquired knowledge, but rather in the way participants expressed this knowledge in the training task. It is conceivable that the participants in the BO-Condition might have noticed the blocked structure of the training blocks and also might have experienced a fluency difference between the random and the regular mini-blocks. This might have helped them to adjust their performance accordingly. When a participant experiences more fluency on the regular trials and less on the deviant trials, they might, for example, be able to focus more on speed on the regular and on accuracy on the deviant trials.

Taken together, the results of Experiment 1 suggest that the different arrangement of the trial types does not affect the amount of implicitly acquired knowledge and therefore provides a solid basis for further investigating whether both groups indeed experience a difference in the subjective feeling of fluency.

## Experiment 2

Experiment 2 tested whether the different arrangement of trials types in the BO- and the RO-conditions affect the experienced subjective feelings of fluency. The findings of Experiment 1 build an important precondition for testing this as they exclude the possibility that a potential difference in experienced fluency can simply be explained by a different amount of acquired knowledge in the two conditions. It is assumed that the blocked arrangement of the trial types leads to perceivable differences in the fluency of deviant and regular trials while the random arrangement should make it unlikely to experience such differences. In order to assess the subjective feeling of fluency, we used the same training phase as in Experiment 1 but a different test phase. Here, participants were asked to compare the subjective fluency of short blocks of regular and deviant trials. If our hypothesis is correct, participants in the BO-Condition should be sensitive to the differences in the fluency of the regular and deviant test trials whereas participants in the RO-Condition should not, or at least to a lesser extent. Since it cannot be excluded that the participants in the BO-Condition adapt to this blocked structure of the test task rather quickly, we also included a control condition in which participants were trained with only random material (All-Random; AR-Condition).

### Method

#### Participants

Sixty students of the University of Cologne (48 women) participated either in fulfillment of course credit or in return for payment, with 20 participants in each of the three conditions, which they were randomly assigned to. The mean age was 23.65 years (range: 17–33; *SD* = 3.68). All participants reported normal or corrected-to-normal vision.

#### Procedure

The training task was designed as described in the General Method Section. There were only two slight differences to Experiment 1. One difference was the stimuli being used; instead of the rather complex colored circles from Experiment 1, we now used simpler colored squares (the colors were: green, pink, cyan, yellow, blue, and red). The other difference was that the training task only consisted of six blocks this time.

In the test task, all participants were asked to compare two consecutive mini-blocks of 18 trials each and rate which of the two blocks felt more fluent to them. One mini-block contained the learned sequence and the other block consisted of random trials only. In order to reach maximal similarity to the sequential trials, the random trials now had the additional constraint that all six positions had to be used, before they could be repeated and no “runs” (e.g., position 1 to 2, 2 to 3, etc.) were allowed. The order of the mini-blocks was random. The first of the mini-blocks was always announced with the notion “1st half” and the second with “2nd half” appearing on the center of the screen. After having responded to the two mini-blocks, participants were asked to rate which of the two halves felt more fluent to them by pressing “1” for the first and “2” for the second half. In total, there were five pairs of such mini-blocks, so participants gave five comparative fluency ratings. The first pair of mini-blocks did not contain any regular trials. It only served to sensitize the participants for experiencing different subjective feelings of fluency: The mini-block that was designed to feel more fluent was structurally equal to the training sequence (i.e., all six keys had to be used before a repetition was allowed, the fingers that had to be used alternated between the left and the right hand). Additionally, the response stimulus containing the target color on trial *t* already appeared on the correct location on trial *t–1*. The mini-block designed to feel less fluent did not have any of these properties and the only constraints were avoidance of repetitions and runs. The data of this block were excluded from further analyses. Besides a general explanation of the test task, participants were instructed to focus on their subjective feeling of fluency and to try not to focus on the factors in the background of the task that might have an influence on this feeling.

If the hypothesis that the participants in the BO-Condition experience differences in the fluency of regular and deviant trials during training, while participants in the RO-Condition do not, is correct, then participants in the BO-Condition should rate the regular mini-blocks as feeling more fluent more often than the RO- or AR-Condition.

### Results

One participant in the AR-Condition had to be excluded because they did not finish the test task. For the remaining 59 participants we analyzed the individual mean error-rates. According to our error criterion of Experiment 1, two participants in the AR-Condition and one participant in the BO-Condition were excluded. For the remaining 56 participants, individual median RTs were computed for each block. Again, we excluded errors and post-error trials (11.43%) as well as RTs > 3000 ms (0.07%) from this computation.

#### Training phase

Tables 3A,B show mean percent error rates and mean of the median RTs for each condition and for each training block. We conducted 2 (Condition) × 6 (Block) × (Sequence Type: regular/deviant) mixed-design ANOVAs for each of the dependent variables to compare the two experimental conditions. In a second step, we then analyzed the difference between these experimental conditions and the AR-Condition with a 3 (Condition) × 6 (Block) mixed-design ANOVA.

**Table 3A:** **Percent error rates and their respective standard deviations (in brackets) by training block, condition, and trial type (regular vs. deviant) in the training phase of Experiment 2**.

**Training Block**	**Random-order condition**	**Random-order condition**	**Blocked-order condition**	**Blocked-order condition**	**All-random condition**
	**Regular trials**	**Deviant trials**	**Regular trials**	**Deviant trials**	**Random trials**
Block 1	5.20 (3.56)	5.93 (3.78)	4.20 (3.65)	6.50 (3.61)	4.86 (3.60)
Block 2	6.51 (4.07)	7.47 (2.76)	5.86 (2.81)	6.56 (4.40)	5.98 (4.61)
Block 3	6.67 (4.12)	8.30 (5.50)	4.80 (2.69)	7.84 (3.22)	5.11 (3.69)
Block 4	6.26 (4.02)	7.80 (4.60)	5.10 (2.24)	6.50 (3.59)	4.57 (3.41)
Block 5	6.10 (4.75)	7.82 (4.17)	5.08 (3.04)	6.84 (3.41)	5.18 (3.87)
Block 6	5.41 (2.80)	7.34 (4.60)	3.91 (2.58)	6.97 (3.92)	6.19 (4.13)

**Table 3B:** **Means of the median RTs and their respective standard deviations (in brackets) by training block, condition, and trial type (regular vs. deviant) in the training phase of Experiment 2**.

**Training block**	**Random-order condition**	**Random-order condition**	**Blocked-order condition**	**Blocked-order condition**	**All-random condition**
	**Regular trials**	**Deviant trials**	**Regular trials**	**Deviant trials**	**Random trials**
Block 1	760.0 (121.8)	766.3 (131.6)	690.3 (63.8)	718.7 (73.9)	736.8 (118.4)
Block 2	766.9 (127.5)	775.5 (120.6)	711.4 (66.6)	732.9 (80.6)	741.6 (118.1)
Block 3	752.0 (119.2)	764.9 (118.2)	689.5 (62.5)	730.4 (67.0)	740.5 (109.3)
Block 4	727.9 (109.9)	740.1 (98.9)	678.1 (65.9)	719.4 (73.4)	718.9 (103.4)
Block 5	725.9 (125.4)	733.5 (114.7)	662.2 (63.1)	701.0 (60.4)	968.5 (73.9)
Block 6	700.7 (106.3)	731.7 (120.8)	657.9 (57.8)	681.7 (61.7)	701.2 (89.8)

For the error rates the first ANOVA revealed a significant main effect of Block [*F*_(5, 185)_ = 3.39, *p* = 0.006, η_*p*_^2^ = 0.084]. This effect could not be attributed to specific differences between single blocks by *post-hoc* tests, though there seemed to be a numerical trend for participants making slightly more errors in the middle of the experiment (lowest mean error rate: Block 1 = 5.7%, highest mean error rate: Block 3 = 7.7%, Block 6 = 6.8%). There also was a significant main effect of Sequence Type, with more errors being made on deviant than on regular trials [*F*_(1, 37)_ = 26.37, *p* < 0.001, η_*p*_^2^ = 0.416]. There were no significant interactions.

The second ANOVA (with all three conditions included) yielded a main effect of Block [*F*_(5, 265)_ = 0.022, *p* = 0.090, η_*p*_^2^ = 0.035; all other *F*s < 1]. *Post-hoc* tests suggest that all conditions showed slightly more errors in the sixth than in the first block [*F*_(1, 53)_ = 3.52, *p* = 0.066, η_*p*_^2^ = 0.062]. There was no Condition × Block Interaction (*F* < 1).

For the RT, the first ANOVA revealed significant main effects of Block [*F*_(5, 185)_ = 28.35, *p* > 0.001, η_*p*_^2^ = 0.434] and Trial Type [*F*_(1, 37)_ = 101.21, *p* < 0.001, η_*p*_^2^ = 0.732]. In addition, the Condition × Trial Type interaction [*F*_(1, 37)_ = 14.19, *p* < 0.001, η_*p*_^2^ = 0.277], and the three-way interaction [*F*_(5, 412)_ = 2.58, *p* = 0.028, η_*p*_^2^ = 0.065] were significant (all other effects *F*s < 1.5, *p* > 0.4).

Thus, the picture of the results is a bit more complex than it was in Experiment 1. As can be seen from Table 3B, the mean RTs in both conditions decreased due to practice and deviants led to generally slower reaction times than regular trials. However, we did not find a significant Block × Trial Type interaction (*F* < 1). This might be partially due to the BO-Condition showing large RT-differences between regular and deviant trials from Block 1 on [*F*_(1, 37)_ = 17.68, *p* < 0.001, η_*p*_^2^ = 0.323] whereas the RO- Condition merely showed a trend of an increasing difference between regular and deviant trials, when comparing Block 1 with Block 6 [*F*_(1, 37)_ = 3.24, *p* = 0.080, η_*p*_^2^ = 0.081]. In parts, these differences between conditions already explain the significant Condition × Trial Type and the Condition × Block × Trial Type interactions. *Post-hoc* comparisons additionally showed that the participants in the BO-Condition responded faster to regular trials than participants in the RO condition [*F*_(1, 37)_ = 3.73, *p* = 0.060, η_*p*_^2^ = 0.092], whereas the deviant trials showed no such difference [*F*_(1, 37)_ = 1.74, *p* = 0.190).

The second ANOVA only showed a main effect for Block [*F*_(5, 265)_ = 24.37, *p* < 0.001, η_*p*_^2^ = 0.315], suggesting a general practice effect for all conditions. No other effect was significant (*F*s < 1.50, *p*s > 0.3).

#### Test phase: reaction times

As a manipulation check, we first computed a 3 (Condition) × 4 (Block) × 2 (Mini-Block: regular/deviant) mixed-design ANOVA with mean RTs in the test phase as dependent variable. The ANOVA yielded a significant main effect of Mini-Block, with regular mini-blocks leading to shorter median RTs than deviant mini-blocks [*F*_(1, 53)_ = 4.12, *p* = 0.047, η_*p*_^2^ = 0.072]. This main effect was qualified by a significant Condition × Mini-Block interaction [*F*_(2, 53)_ = 8.85, *p* < 0.001, η_*p*_^2^ = 0.250] and a significant Condition × Block × Mini-Block interaction [*F*_(6, 159)_ = 2.46, *p* = 0.0264, η_*p*_^2^ = 0.085]. Additionally, the Block × Mini-Block interaction was significant [*F*_(3, 159)_ = 3.31, *p* = 0.022 η_*p*_^2^ = 0.059; all other *F*s < 1].

As can be seen from Table 4, the first two interactions were mainly due to different performance patterns in the AR- vs. the BO- and the RO-Conditions. Participants in the AR-Condition showed faster median RTs for the deviant mini-blocks than for the regular mini-blocks [*F*_(1, 53)_ = 4.81, *p* = 0.033, η_*p*_^2^ = 0.083]. By contrast, participants in the RO- and the BO-Conditions showed the expected reversed pattern [faster responses for regular than for random trials: *F*_(1, 53)_ = 12.05, *p* = 0.001, η_*p*_^2^ = 0.185, *F*_(1, 53)_ = 6.22, *p* = 0.016, η_*p*_^2^ = 0.105 for the RO- and the BO-Condition, respectively]. They did not differ significantly from each other (*F* < 1). This resembles the results of the transfer test of Experiment 1 and strengthens the assumption that both conditions developed at least some sequence knowledge. In the RO-Condition, this difference between mini-block type increased with practice showing larger RT-differences in the last two blocks than in the first two blocks [*F*_(1, 53)_ = 10.05, *p* = 0.002, η_*p*_^2^ = 0.159]. This was not true for the BO-Condition, which showed comparable differences between these blocks (*F* < 1). Hence, it could be assumed that participants in the RO-Condition needed some time to adapt their behavior to the new structure of the material.

**Table 4:** **Means of the median RTs and their respective standard deviations (in brackets) by training block, condition, and trial type (regular vs. deviant) in the test phase of Experiment 2**.

**Test mini-block**	**Random-order condition**	**Blocked-order condition**	**All-random condition**
Regular 1	708.4 (112.6)	634.0 (56.4)	684.3 (106.9)
Deviant 1	716.1 (139.9)	640.6 (72.6)	651.3 (73.2)
Regular 2	696.2 (131.2)	624.2 (56.0)	679.6 (107.6)
Deviant 2	701.8 (118.6)	655.9 (57.2)	657.3 (78.6)
Regular 3	671.0 (114.3)	648.9 (78.3)	663.8 (101.8)
Deviant 3	731.8 (117.3)	653.9 (75.2)	662.4 (101.8)
Regular 4	691.8 (122.6)	621.9 (69.0)	658.0 (108.7)
Deviant 4	716.9 (138.9)	651.9 (62.5)	656.5 (76.4)

Because Experiment 2 has no test for the learning effects on its own and mostly relies on the interpretation of Experiment 1, the non-significant difference between the RTs of the BO- and the RO-Condition in this test phase is particularly important. Therefore, we calculated a Bayes factor with the same estimated maximum effect of the transfer test of Experiment 1 [*B*_*H*(0.40 *ms*)_ = 0.30]. Though not specifically designed to test the participants' knowledge, the test phase of Experiment 2 can help to support the assumption that the BO- and the RO-Condition do not substantially differ in their acquired knowledge.

#### Test phase: preference judgements

To test whether the arrangement of the trial types in the training task influenced the perceived fluency of the task, we analyzed how often the regular or the deviant mini-block was chosen as feeling more fluent. A 2 (Fluency-Judgement: regular/deviant) × 3 (Condition) Chi-Square Test showed a significant difference between the conditions [χ_(2)_ = 7.46, *p* = 0.02, *V* = 0.09]. Most important for our hypotheses, the RO- and the BO-Condition differed significantly [χ_(1)_ = 3.92, *p* = 0.04, φ = 0.16]. The BO-Condition rated the regular sequence as feeling more fluent more often (see Table 5). The RO- and AR-Condition did not differ in their choices (*p* = 0.45).

**Table 5:** **Frequencies of sequential and random mini-blocks rated as feeling more fluent in the test phase of Experiment 2**.

**Mini-block**	**Random-order condition**	**Blocked-order condition**	**All-random condition**
Sequential	39	49	29
Random	41	27	39

In addition, we also tested the relation between preference judgements and the mean RT-differences. If participants rely their judgement about the feeling of fluency on RT-differences between regular and the random mini-blocks (median RT deviant mini-block—median RT regular mini-block), we should find a positive correlation between preference judgements and RT-differences. Indeed, this correlation was positive (*r* = 0.22, *p* < 0.05).

### Discussion

Overall, Experiment 2 confirmed our main hypothesis that participants in the BO-condition developed a stronger sensitivity for the fluency differences between the random and the regular material than the RO condition. However, the analysis of the median RTs of the training phase showed some unexpected patterns. Only the RO-Condition showed a trend toward a Block × Trial Type interaction, while the BO-Condition showed a significant difference between random and regular trials from Block 1 on. We have no really convincing explanation for this unexpected finding as we used, with the exception of the presented stimuli and the somewhat shorter training phase, exactly the same training phase as in Experiment 1. Since participants' knowledge was not assessed in Experiment 2, the interpretation of the reaction time data is difficult. Even though the reaction times in the RO- and BO-Conditions seem to suggest that knowledge about the sequence has been acquired, the data are not very clear cut and it cannot fully be excluded that both conditions did learn the sequence to a different extent. However, the results of Experiment 1 suggested that both conditions did not differ in the extent of learning, but differed in the expression of this learned knowledge during training. Like already suggested by a statistical trend in Experiment 1, there was a significant Condition × Trial Type interaction in Experiment 2. Therefore, replicating this performance difference between the BO- and the RO-Condition is an interesting finding. These preliminary considerations seem to be supported by the reaction times of the test task. Comparable to the transfer test in Experiment 1, the RO- and the BO-Condition showed significant and comparable differences between regular and deviant mini-blocks as the material changed to a blocked arrangement for all participants. Additionally, the RO-Condition showed larger differences between regular and deviant trials in the last two blocks of the test task, which could mean that this group needed a few trials to adjust to the blocked arrangement until their performance matched the performance of the BO-Condition. The AR-Condition did not show any adjustment to the material over the four test blocks.

Most important to our hypothesis, the BO-Condition did rate the regular mini-blocks as feeling more fluent than the deviant mini-blocks. This was not the case in the RO-Condition. It seems that the training with the blocked material leads to the development of a higher sensitivity toward the differences in the experienced fluency of deviant and regular trials. Additionally, we found a correlation between the reaction time differences and the tendency to rate the regular mini-blocks as feeling more fluent. This might be a hint that the RTs provide a basis for the feeling of fluency, with faster RTs being experienced as being more fluent. However, it is also possible that the feeling of fluency relies on other cues like the feeling of a repeating movement. Alternatively, the feeling of fluency might also influence the reaction times as suggested by the observed Condition × Trial Type interactions.

## Experiment 3

Experiments 1 and 2 demonstrated that the different arrangements of the training material did not affect the extent of acquired implicit learning, but it did affect the sensitivity toward the differences in the experienced fluency between regular and deviant trials. According to the UEH these (consciously) perceivable differences in the fluency of the task are a violation of the expectancies of a participant who concurrently perceives no differences on the surface of the task. This unexpected violation of expectancies triggers an attributional process which in turn can lead to explicit knowledge of the underlying sequence. Therefore, Experiment 3 is designed to test whether the blocked arrangement of the different trial types leads to more conscious knowledge than the random arrangement. In Experiment 1, we already tested for explicit knowledge using the Wager Task. This task did not reveal any signs of explicit knowledge. We assume that the short intervals (22 trials each) make it difficult for explicit search processes to successfully result in finding the sequence as any traces of emerging explicit knowledge are rejected quickly because of the following deviant trials. Therefore, we decided to prolong the intervals of regular and deviant trials in the BO-Condition to make it easier for the participants to detect the underlying sequence.

### Method

#### Participants

Sixty-four students of the University of Cologne (39 women) participated either in fulfillment of course credit or in return for payment. Their mean age was 24.50 years (range: 18–44, *SD* = 5.0). All participants reported normal or corrected-to-normal vision. Thirty-two participants were in the BO-Condition and 32 participants in the RO-Condition^1^.

#### Procedure

The training task in Experiment 3 was identical to the training task of Experiment 1 with two exceptions. First, the mini-blocks of regular and deviant trials now contained 88 instead of 22 trials each. Second, the training task lasted eight instead of seven blocks. The stimuli again were the more complex stimuli of Experiment 1. Again, all participants received the wager task of Experiment 1 after training.

### Results

Two participants in the RO-Condition had to be excluded, due to technical problems with the computers.

For the remaining 62 participants we analyzed the individual mean error-rates. Our criterion led to the exclusion of two participants in the RO-Condition and one in the BO-Condition. For the remaining participants individual median RTs were computed for each block. We excluded errors, post-error trials (12.30%) and RTs > 3,000 ms (0.33%) from this computation^1^.

#### Training phase

Tables 6A,B present the mean percent error rates and the means of the median RTs per condition, block, and trial type. We conducted 2 (Condition) × 8 (Block) × (Sequence Type: regular/deviant) mixed-design ANOVAs separately for the two dependent variables.

**Table 6A:** **Percent error rates and their respective standard deviations (in brackets) by training block, condition, and trial type (regular vs. deviant) in Experiment 3**.

**Training block**	**Random-order condition**	**Random-order condition**	**Blocked-order condition**	**Blocked-order condition**
	**Regular trials**	**Deviant trials**	**Regular trials**	**Deviant trials**
Block 1	8.79 (4.46)	9.57 (5.97)	6.33 (4.29)	7.48 (4.82)
Block 2	6.56 (4.18)	7.80 (4.55)	5.77 (3.64)	6.49 (3.79)
Block 3	5.73 (3.81)	8.10 (4.00)	4.53 (3.79)	7.01 (4.62)
Block 4	6.03 (4.76)	7.75 (4.64)	5.07 (4.62)	6.09 (3.50)
Block 5	5.87 (3.86)	7.50 (5.71)	5.40 (3.72)	5.96 (3.81)
Block 6	5.20 (3.76)	6.27 (4.98)	5.15 (5.68)	6.56 (4.11)
Block 7	5.27 (4.46)	8.30 (6.49)	5.78 (4.50)	6.05 (3.98)
Block 8	6.75 (4.98)	7.74 (4.27)	5.63 (3.78)	7.18 (5.33)

**Table 6B:** **Means of the median RTs and their respective standard deviations (in brackets) by training block, condition, and trial type (regular vs. deviant) in Experiment 3**.

**Training block**	**Random-order condition**	**Random-order condition**	**Blocked-order condition**	**Blocked-order condition**
	**Regular trials**	**Deviant trials**	**Regular trials**	**Deviant trials**
Block 1	955.3 (166.1)	963.2 (155.0)	904.3 (151.4)	927.4 (164.2)
Block 2	893.4 (108.8)	911.7 (116.4)	859.1 (128.9)	879.0 (125.5)
Block 3	875.7 (104.3)	887.9 (100.2)	808.4 (127.6)	858.5 (107.2)
Block 4	835.7 (92.4)	868.1 (86.4)	786.0 (147.5)	836.0 (98.0)
Block 5	815.4 (80.7)	849.4 (76.8)	765.0 (157.6)	816.0 (96.2)
Block 6	822.2 (77.7)	840.9 (85.7)	731.1 (152.9)	802.2 (93.2)
Block 7	796.2 (80.8)	825.6 (97.6)	723.7 (179.6)	794.1 (76.9)
Block 8	790.9 (85.4)	812.5 (87.1)	711.9 (167.4)	792.2 (89.6)

For the error rates, the ANOVA yielded a significant main effect of Block [*F*_(7, 399)_ = 4.78, *p* < 0.001, η_*p*_^2^ = 0.463], and of Sequence Type [*F*_(1, 57)_ = 42.31, *p* < 0.001, η_*p*_^2^ = 0.415]. The effect of Block indicated that participants in both conditions made fewer errors over the course of training. The main effect of Sequence Type was caused by higher error rates for deviant than for regular trials. No interaction reached the level of significance.

Concerning the median RTs the ANOVA revealed significant main effects of Block [*F*_(7, 399)_ = 74.64, *p* < 0.001, η_*p*_^2^ = 0.557] and of Trial Type [*F*_(1, 57)_ = 21.50, *p* < 0.001, η_*p*_^2^ = 0.274]. Participants responded faster over the course of the training and were generally slower on deviant trials. In addition, the Block × Trial Type interaction [*F*_(7, 399)_ = 2.42, *p* = 0.020, η_*p*_^2^ = 0.041] was significant, showing that the difference between regular and deviant trials increased over the course of training. *Post-hoc* tests suggest that the Block × Trial Type interaction was mainly caused by the BO-Condition showing a large difference between the first and the last block [*F*_(1, 57)_ = 5.60, *p* = 0.021, η_*p*_^2^ = 0.090], whereas the RO-Condition surprisingly did not show such a difference (*F* < 1). This, again could be due to the RO-Condition either gaining no knowledge about the sequence or because the arrangement of trial types made it difficult to express sequence knowledge. No other interaction reached level of significance.

#### Test phase: wager task

As in Experiment 1, we first tested if the participants in the two conditions gave more correct predictions than was expected by chance (20% correct predictions). This was especially important as the participants in the RO-Condition did not show a significant learning effect during the SRT training. Both conditions showed significantly more correct predictions than expected by guessing [*t*_(27)_ = 1.74, *p* = 0.05, one-tailed, for the RO-Condition; *t*_(30)_ = 4.24, *p* < 0.001, one-tailed, for the BO-Condition]. Thus, the wager task revealed that both conditions acquired a significant amount of sequence knowledge. As in Experiment 1, this finding suggest that the RO-Condition did not express their acquired knowledge in the same way as the BO-Condition did in the training task.

Different to Experiment 1 but in accordance with our hypothesis, the BO-Condition gave significantly more correct predictions than the RO-Condition [RO-Condition: 23%, BO-Condition: 42%; *t*_(57)_ = 3.21, *p* < 0.001]. Albeit, both conditions received the same amount of regular trials, the BO-Condition acquired much more knowledge about the sequence than the RO-Condition.

The second analysis aimed to test if this larger amount of knowledge in the BO-Condition concerns only implicit or also explicit knowledge (see Table 7). A 2 (Condition) × 2 (Wager Type: high|correct vs. high|false) mixed design ANOVA yielded no significant main effect for Condition, implying that no condition had a greater tendency to give high wagers (*F* < 1). The main effect for Wager Type was significant and showed that more high wagers were given when the prediction was correct [*F*_(1, 57)_ = 5.87, *p* = 0.019, η_*p*_^2^ = 0.093]. Most importantly, there was a significant Condition × Wager Type interaction [*F*_(1, 57)_ = 4.61, *p* = 0.036, η_*p*_^2^ = 0.075], showing that the participants in the BO-Condition were better able to use their knowledge strategically. *Post-hoc* tests further showed that only the participants in the BO-Condition put more high wagers on correct than on false predictions [*F*_(1, 75)_ = 11.01, *p* = 0.002, η_*p*_^2^ = 0.162, RO-Condition: (*F* < 1)]. This implies that the participants in the BO-Condition possessed more explicit knowledge than the participants in the RO-Condition.

**Table 7:** **Percent correct predictions, percent high wagers given when the prediction was correct, percent high wagers given when the prediction was false, and their respective standard deviations (in brackets) by condition in Experiment 3**.

**Condition**	**Percent correct predictions**	**Percent high wager| correct prediction**	**Percent high wager| false prediction**
Random-order	23.02 (9.16)	59.75 (30.95)	58.70 (30.52)
Blocked-order	41.45 (29.07)	65.78 (30.06)	48.43 (36.00)

Like in Experiment 1, we wanted to exclude that the participants acquired explicit knowledge during the test task. Therefor we contrasted the difference between high|correct and high|false wagers in the first 12 wager trials with that of the last 12 wager trials for both conditions. No Condition showed a significant increase in the difference between high|correct and high|false wagers [RO-Condition: *t*_(27)_ = 1.38, *p* = 0.178; BO-Condition: *t*_(30)_ = 1.12, *p* = 0.271].

### Discussion

Experiment 3 provided one important result: Prolonging the mini-blocks from 22 trials to 88 trials led to explicit knowledge in the BO-Condition, even though the number of sequence trials was not increased. This finding comes along with one weakness, however. The data of the training did not reveal any sequence learning in the RO-Condition (even after increasing the number of participants). Only the wagering task suggested that participants in this condition did possess some implicit sequence knowledge.

Analyzing the reaction times of the training task could only confirm a learning effect for the BO-Condition while the RO-Condition did not show any signs of sequence learning in their median RTs. As argued before, this could either mean that the RO-Condition did not acquire any knowledge of the training sequence or that they controlled their behavior differently than the participants in the BO-Condition. Because the participants in the RO-Condition never experience longer episodes of regular trials where they can express their knowledge fluently, they might not show their knowledge in their overt behavior and act more stimulus-dependent. This is also the only experiment in this series that could not reveal any signs of a learning effect in the reaction times of the RO-Condition, possibly hinting at a power problem. Supportive for the assumption that the participants in both conditions did acquire some sequence knowledge, the wager task showed that both groups were able to make more correct predictions than to be expected by mere guessing. This is important for further testing our hypothesis that it is the difference in the experienced fluency that leads to differences in the generation of explicit knowledge rather than the difference in the strength of associative weights acquired in the training task. Still, as Experiment 3 was designed to show differences in acquired explicit knowledge, we are not able to show that the implicit knowledge base is comparable in both conditions because if the BO-Condition indeed did acquire more explicit knowledge, this will also lead to more correct predictions in the wager task. This is essentially what we found, analyzing the wager task. Not only did the BO-condition make far more correct predictions about the next response, they also were able to use this knowledge strategically. Giving more high wagers when a prediction was correct while giving less high wagers when a prediction was false indicates that the participants not only developed first-order knowledge about the sequence but also second-order knowledge of knowing if they knew or did not know the correct prediction. Taken together, Experiment 3 showed that being trained with the blocked arrangement of the trial types leads to more explicit knowledge than being trained with the random arrangement of the trial types.

## General discussion

It was the aim of this article to test two important theories on the generation of explicit knowledge in an implicit learning situation against each other. Single-system accounts on the one hand, respectively strengthening views, assume a gradual difference between unconscious, implicit and conscious, explicit knowledge (Cleeremans, 2008, 2011; Timmermans et al., 2012). Multiple-system theories on the other hand disagree with the idea that representational strength is a sufficient factor to explain the transition from implicit to explicit knowledge (Sun et al., 2001; Frensch et al., 2003; Scott and Dienes, 2008). Here we focused on one of these multiple-system theories, namely the Unexpected Event Hypothesis (Frensch et al., 2003; Haider and Frensch, 2005, 2009; Rünger and Frensch, 2008). The UEH assumes that implicit learning leads to changes in behavior which can be consciously perceived as unexpected changes. Once an unexpected change in one's own behavior is observed, attributional processes can lead to the generation of explicit knowledge. Various unexpected events, here the unexpected difference in the experienced subjective fluency, can serve as a trigger to start these attributional search processes. The UEH views consciousness as an absolute, dichotomous state, resulting from sudden insight rather than a gradual developing state.

To test the UEH against strengthening accounts, we aimed to create an experimental situation in which two groups should not differ in their acquired associative strength because they experienced the same amount of correct and incorrect sequence transitions during a training task. The only difference between the two groups was supposed to be in their opportunities to experience differences in their feelings of fluency due to the different arrangement of the regular and deviant trials. If a simple strengthening account was correct, both groups should show a comparable amount of explicit knowledge. If instead the assumption that it needs an unexpected event to develop explicit knowledge was correct, the group that experiences differences in their feelings of fluency should develop more explicit knowledge. In Experiment 1 we were able to show that the difference in the arrangement of regular and deviant trials led to comparable amounts of acquired knowledge, demonstrated both in a transfer and in a wager task as well. This knowledge seemed to be mainly implicit, as the wager task revealed that participants of both conditions were unable to strategically use their knowledge. With Experiment 2, we were further able to show that the differences in the arrangement of the trial types led to the predicted differences in the feeling of fluency between the groups. Together these results provided the necessary preconditions for Experiment 3 in which we aimed to show that these differences in the experienced fluency eventually lead to differences in the resulting explicit knowledge. Experiment 3 demonstrated that the participants in the BO-Condition generated more explicit knowledge than the participants in the RO-Condition.

Taken together, these results favor the UEH over a simpler strengthening account. We assume that in our experiments it is the difference of fluency between regular and deviant trials, which can only be experienced in the BO-Condition that serves as an unexpected event. Our results fit nicely with a recently published study by Yordanova et al. (2015) who found that participants who gain explicit knowledge show a greater variability in their RTs which the authors interpret as an unexpected mismatch that may serve as an offline trigger for explicit learning processes.

Nevertheless, there are several critical empirical points in our experiment that need further discussion and subsequent research. First of all, we could not assume comparable performances in the training as the reaction times differed between the BO- and the RO-Condition. The interpretation of our data would be simpler and more clear-cut if the reaction times of Experiments 1 and 2 could support the assumption that both conditions do not differ in their acquired knowledge. Instead, for all three experiments, the RT-difference between regular and deviant trials was smaller in the RO- than in the BO-Condition. In fact, Experiment 3 even failed to show a significant Block × Trial Type contrast for the RO-Condition. If these results are interpreted as showing that the RO-Condition learned less about the sequence, the interpretation of the remaining results would be ambiguous, favoring the more parsimonious strengthening account.

However, due to the design of the training, the knowledge tests in Experiment 1 and 3 seem to provide more reliable information about the extent of sequence knowledge. Different studies have shown that the performance in an SRTT can be an unreliable measure of sequence knowledge, as it can, for example, vary with different instructions for speed or accuracy (Hoyndorf and Haider, 2009), with perceived conflict (Jiménez et al., 2009), or task demands (Frensch et al., 1999). It seems plausible to assume that the differences in experienced fluency are accompanied by differences in performance during the training. It might be that in the BO-Condition, the more fluent experience on regular trials leads to a focus on speed while the less fluent deviant trials favor a focus on accuracy. Complementary, in the RO-Condition participants might show less decreased RTs on regular trials as the discontinuous structure of the task leads to an extenuated expression of the knowledge. The transfer test in Experiment 1 supports this argument as the BO- and the RO-Conditions show the same performance once the structure of the task was identical for both conditions.

Another aspect that has to be discussed is the representational structure of the implicitly learned sequences. Because the BO- and the RO-Conditions differ in the arrangement of the trial types, it is possible that the representational structures differ between the two groups. It is conceivable that the participants in the BO-Condition learn something about the embedded, predictable structure of the training task (Cleeremans and McClelland, 1991; Pacton et al., 2015). Progressing in training, they might learn that there is a deterministic structure embedded after every 22 unpredictable trials and cognitively separate regular and deviant chunks. From then on, the irregular trials would no longer weaken the associative strength of the sequential weights, leading to a stronger sequential representation in the BO- then in the RO-Condition. While further experiments should be conducted to exclude this explanation, we consider it to be the less likely explanation. Research has shown that embedded random sequences can have a weakening effect on the associative weights before the system starts to learn that there is a certain predictive structure in the task and adjusts to this (Cleeremans, 1993). With six to eight blocks, our training task is most likely too short to learn about this predictive structure. Also, again, the data of our test tasks in Experiment 1 seem to speak against the assumption that the participants in the BO- and the RO-Condition have learned the sequence to a different extent.

A third objection might address the results of Experiment 3 which showed that there was more explicit knowledge when the participants were trained with a blocked arrangement. It could be argued that, due to the same strength of implicitly learned sequence knowledge, both groups did try to find a sequence, but only the BO-Condition could be successful in their search, while the RO-Condition desisted from the idea of a possible underlying sequence because the probabilistic structure made it too difficult to find it. This assumption can be supported by the literature comparing probabilistic and deterministic sequence learning. Various authors have demonstrated that a probabilistic sequence does interfere with the acquisition of explicit knowledge. When participants are informed in advance of the training that there is a certain structure or even what this structure is, they are not able to profit from this information and usually show no sign of explicit sequence knowledge (Schvaneveldt and Gomez, 1998; Stefaniak et al., 2008). However, research on probabilistic sequence learning uses this special kind of structure for suppressing explicit learning without addressing the question why it usually does not lead to explicit knowledge. It seems to be the a priori assumption that additional explicit learning processes cannot be successful under probabilistic conditions; this could have been the case in our experiments as well. Alternatively, it is possible that probabilistic learning conditions lead to weaker representations because (a) regular trials are often not equalized between deterministic and probabilistic conditions and (b) even when they are, deterministic conditions lack the weakening effect of additional deviant trials. Our BO-Condition, especially in Experiment 3, might be seen as a deterministic structure interrupted by blocks of deviant trials. Taken together with Experiment 1, our results speak against the role of representational strength for differences in the acquired explicit knowledge between probabilistic and deterministic sequences. This leaves two further explanations: First, as stated above, participants do in fact acquire enough strength to develop explicit knowledge but cannot further strengthen this knowledge because the probabilistic structure is too difficult for an explicit learning process. However, this option is not entirely compatible with a simple strengthening account, as the explicit knowledge should develop depending only on the strength of associations. An additional explicit learning process would have to be assumed that is triggered by representational strength but does not rely on it itself. The second option, in line with our hypothesis, is that the probabilistic structure hinders any metacognitive judgements about one's own behavior during the training task. Therefore, there is no situation where a triggered explicit learning process fails due to the complex structure of the task. A preliminary argument for the second option would be the lack of explicit knowledge at the end of the wager task. If participants had an idea about an underlying sequence during training it seems most likely that, as soon as the wager task starts, they remember their initial assumption and start to search for the sequence again. The data for the RO-Condition showed no signs of developing explicit knowledge during the wager task. Additionally, it would have to be explained why the participants in the RO-Condition could not discriminate between the fluency of regular and deviant mini-blocks in the test of Experiment 2, if both conditions did not differ in their experienced fluency during training. Finally, it has to be mentioned that we do not disagree with the idea that even if we told the participants in the RO-Condition that there was a sequential structure, they would not be able to find it. Our main point is that any additional explicit learning process is not triggered directly by associative strength and that, maybe even more importantly, associative strength of the transitions does not govern explicit learning processes.

Also, the current experiments are not designed to test the finer predictions of the UEH, like, for example, the assumption that the explicit attributional processes have no direct access to the implicitly learned knowledge. However, while further experiments are certainly needed to precisely test these assumptions, the current study provides another important empirical piece in the puzzle of how explicit knowledge can develop from implicit knowledge. The results so far are difficult to reconcile with a simple explanation that relies on the strengthening of associative weights and rather appear to be in favor of the UEH. We also believe that the way we manipulated the arrangement of regular and deviant trials with the intention to keep the ratio of regular and deviant transitions balanced has not been used in implicit learning research so far, but might be an interesting methodological addition for studying present controversies in this area of research.

### Conclusion

(1) Experiment 1 demonstrated that our method of manipulating the arrangement of regular and deviant trials does not lead to a difference in the performance in either a transfer test or a wager task. We interpret this as an indicator that the different arrangements do not lead to a difference in the acquired associative strength. (2) Experiment 2 further showed that the participants in the BO-Condition seemed to experience a difference in the fluency of random and regular trials, while the participants in the RO-Condition experienced no such difference. (3) When the intervals of regular and random trials were prolonged in Experiment 3, the BO-Condition demonstrated more explicit knowledge in a wager task. We interpret this difference being caused by the difference in experienced fluency. (4) Taken together, these results seem to be difficult to reconcile with a simple strengthening account of the emergence of explicit sequence knowledge. Rather, a metacognitive account that pronounces the role of unexpected, observable changes in one's own behavior (i.e., experienced differences in fluency) seems to be adequate for explaining the results.

## Ethics statement

For the participation in behavioral studies which are not expected to create stress or harm to the participants no special permission is required in Germany, so no approval from an institutional review board was necessary. All participants gave written informed consent and all procedures were performed in full accordance with German legal regulations and the ethical guidelines of the DGPs (Deutsche Gesellschaft für Psychologie; German Society for Psychology).

## Author contributions

SE: Conception and design of the work, conducting the experiments, data analysis and interpretation, writing of the article. HH: Conception and design of the work, data analysis and interpretation, writing, and revising the article.

## Funding

HH was supported by a grant from the German Research Foundation (HA-5447/10-1).

### Conflict of interest statement

The authors declare that the research was conducted in the absence of any commercial or financial relationships that could be construed as a potential conflict of interest.

## Footnotes

^1^We would like to mention that we initially only had 22 participants in each condition and later added 10 more to each condition. The reason is that we failed to find a significant learning effect in the wager task of the RO-Condition after the first 22 participants. Because the learning effect under the RO-Condition was present in Experiment 1 where we had more participants, and because a learning effect was marginally present after the first twenty-two participants, we decided to collect more data (for a debate about the pros and cons of this practice see: Simmons et al., 2011; Murayama et al., 2014).
